# Modulatory Effect of Chlorogenic Acid and Coffee Extracts on Wnt/β-Catenin Pathway in Colorectal Cancer Cells

**DOI:** 10.3390/nu14224880

**Published:** 2022-11-18

**Authors:** Hernán Villota, Gloria A. Santa-González, Diego Uribe, Isabel Cristina Henao, Johanna C. Arroyave-Ospina, Carlos J. Barrera-Causil, Johanna Pedroza-Díaz

**Affiliations:** 1 Grupo de Investigación e Innovación Biomédica, Facultad de Ciencias Exactas y Aplicadas, Instituto Tecnológico Metropolitano, Medellín 050012, Colombia; 2 Productos Naturales Marinos, Facultad de Ciencias Farmacéuticas y Alimentarias, Universidad de Antioquia UdeA, Medellín 050010, Colombia; 3 Department of Gastroenterology and Hepatology, University Medical Center Groningen, University of Groningen, 9713 GZ Groningen, The Netherlands; 4 Grupo de Investigación Davinci, Facultad de Ciencias Exactas y Aplicadas, Instituto Tecnológico Metropolitano, Medellín 050034, Colombia

**Keywords:** chlorogenic acid, coffee extracts, colorectal cancer, Wnt/β-catenin pathway, therapeutic targets

## Abstract

The Wnt/β-Catenin pathway alterations present in colorectal cancer (CRC) are of special interest in the development of new therapeutic strategies to impact carcinogenesis and the progression of CRC. In this context, different polyphenols present in natural products have been reported to have modulatory effects against the Wnt pathway in CRC. In this study, we evaluate the effect of two polyphenol-rich coffee extracts and chlorogenic acid (CGA) against SW480 and HT-29 CRC cells. This involved the use of MTT and SRB techniques for cell viability; wound healing and invasion assay for the evaluation of the migration and invasion process; T cell factor (TCF) reporter plasmid for the evaluation of transciption factor (TCF) transcriptional activity; polymerase chain reaction (PCR) of target genes and confocal fluorescence microscopy for β-Catenin and E-Cadherin protein fluorescence levels; and subcellular localization. Our results showed a potential modulatory effect of the Wnt pathway on CRC cells, and we observed a reduction in the transcriptional activity of β-catenin. All the results were prominent in SW480 cells, where the Wnt pathway deregulation has more relevance and implies a constitutive activation of the signaling pathway. These results establish a starting point for the discovery of a mechanism of action associated with these effects and corroborate the anticancer potential of polyphenols present in coffee, which could be explored as chemopreventive molecules or as adjunctive therapy in CRC.

## 1. Introduction

Polyphenols are natural organic compounds of particular interest in nutrition and functional food [1]. Dietary polyphenols play an important role in human health by regulating cellular metabolism, chronic disease, and cell proliferation [2,3]. To date, several polyphenols have been identified in different natural products, such as coffee which contains phenolic acids such as chlorogenic, caffeic, and feluric [4]; however, their impact on human health has not been fully characterized. Polyphenols from coffee regulate different biological processes, exhibiting chemopreventive, antioxidant, anti-inflammatory, and anticancer properties in in vitro and in vivo studies [5]. Some epidemiological studies also suggest that regular coffee consumption influences the prevention of cardiovascular diseases, obesity, diabetes, and some types of cancer [6,7].

Colorectal cancer (CRC) is one of the most frequent cancers globally, occupying third place after lung and prostate cancer in men and breast cancer in women. Additionally, 10% of all newly diagnosed cases and cancer-related deaths are associated with CRC [8]. Age, genetic and environmental factors play an important role in the development of CRC, and some studies have established a range of hereditary for CRC from 12% to 32%, suggesting that a large proportion of cases (68–82%) have modifiable risk factors, such as overweight and obesity, alcohol consumption, and processed meat intake, among others [9].

The development of CRC is strongly influenced by hereditary factors such as familial adenomatous polyposis (FAP) or Lynch syndrome; however, approximately 80% of cases are sporadic. The events leading to CRC develop slowly through a sequential progression and are staggered. Two pathways of CRC initiation have been described, the adenoma-carcinoma sequence, related to 60–85% of cases, and the alternative, or serrated, pathway, related to 15–40% of cases. These models include mutations in signaling pathways such as Wnt, mitogen-activated protein kinases (MAPK), transforming growth factor-beta (TGF-β), and p53. Tubular, tubulovillous, and villous adenomas are the most common lesions related to sporadic tumors in the adenoma-carcinoma sequence, where the most frequent molecular alterations are related to mutations in the adenomatous polyposis coli (APC) gene. On the other hand, the “serrated neoplastic pathway” describes the progression of serrated polyps, including sessile serrated adenomas and traditional serrated adenomas, with colorectal cancer. This pathway is associated with β-Catenin, BRAF mutation, and microsatellite instability, triggering CRC phenotypes such as CpG island methylator phenotype (CIMP) [10,11].

**Figure 1:**
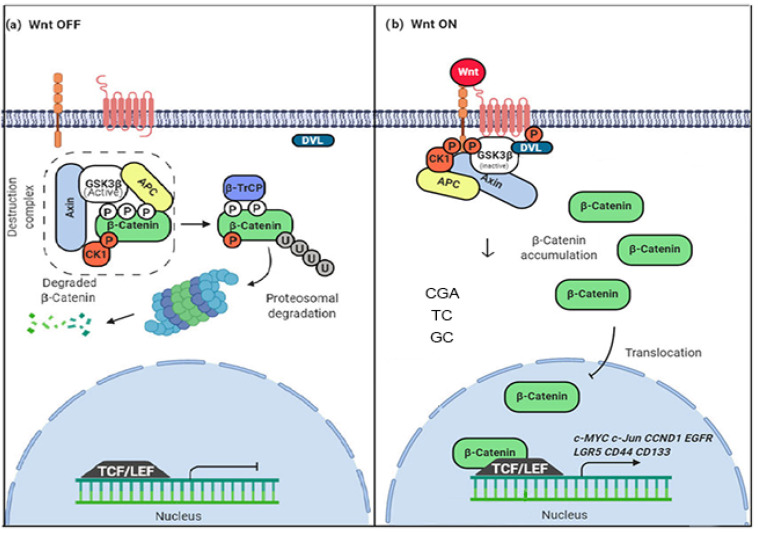
Mechanism of modulation of the Wnt/β-catenin pathway by coffee polyphenols. (**a**) Normal OFF state. The β-catenin degradation complex regulates the levels of β-catenin and controls its transcriptional activator activity (**b**) The normal ON state. The β-catenin degradation complex doesn’t work, β-catenin levels increase, and translocation to the nucleus possibility its transcriptional activity. The possible mechanism of modulation associated with coffee polyphenols; CGA (Chrologenic acid), TC (Toasted coffee), and GC (Gree coffee) decrease the levels of β-catenin in the cytoplasm and nucleus, possibly through a β-catenin degradation mediation and nuclear translocation inhibition. Figure adapted from [14].

The Wnt pathway, widely studied for its importance in processes of embryogenesis, tissue homeostasis, and cell-cell adhesion, plays a crucial role in the initiation, progression, and metastasis of CRC. Wnt pathway activation depends on the alteration of its components and their functions. The transduction of the pathway includes processes such as the secretion of Wnt proteins, identification of Wnt co-receptors, silencing of the β-catenin destruction complex that includes proteins such as (APC and GSK3-β), translocation of β-catenin to the nucleus, recruitment of cofactors, and transcriptional activation of genes such as Cyclin D1 (*CCND1*), *MYC*, and *JUN* (Figure 1). Some aberrant functions in these processes can influence the development of cancer. In CRC, it has been shown that about 90% of cases are a consequence of damage to one of these Wnt pathway processes, especially APC loss-of-function mutations, β-catenin activation mutations leading to hyperactivation, and increased frizzled family receptors. However, APC and β-catenin mutations are generally mutually exclusive, with somatic APC mutations being found in more than 80% of sporadic colorectal tumors and β-catenin mutations in about 48% of tumors without APC mutation [12,13].

Recently, some studies have shown the anticancer activity of different polyphenols present in natural products such as grapes, apples, berries, herbs, spices, and drinks such as coffee. In colorectal cancer specifically, polyphenols show the capability of modulating the Wnt/β-catenin pathway using different regulatory mechanisms such as the decreased phosphorylation of GSK3-β by silibinin and epigalocatequin galato (EGCG) and destabilization of the β-catenin/TCF complex by resveratrol [14]. Coffee is the main natural source of chlorogenic acid (CGA), an hydroxycinnamic acid that is the most abundant polyphenol in natural products. Several in vitro and in vivo studies using CGA have shown its antioxidant activity, inhibition of mutagenic and carcinogenic *N*-nitroso compounds, DNA damage inhibition, and suppression of reactive oxygen species-mediated nuclear factor activation (NF-κB) [15]. Regarding the modulatory effect of CGA, some in vitro and in vivo studies have shown effects related to the regulation of the Wnt pathway, for example, inhibition of adipogenesis in murine fibroblast cells [16], inhibition of cell differentiation in human pulp stem cells [17], and antitumor activity in murine breast cancer models [18]. Additionally, we recently reported the biological effect of CGA and caffeic acid related to the inhibition of cell migration as a first approach to the study of the effect of polyphenols present in coffee in colorectal cancer models at the in vitro level [19]. However, the modulatory effect of coffee polyphenols on the Wnt pathway has not been studied in CRC models. For these reasons, in the present study, we explore the possible modulation of the Wnt/β-catenin pathway by CGA and two coffee extracts rich in polyphenols to evaluate the influence of treatments on cell proliferation, gene expression, Wnt transcriptional activity, and β-catenin and E-cadherin subcellular localization, using SW480 and HT-29 colorectal cancer cell lines.

## 2. Materials and Methods

### 2.1. Chemical and Reagents

Neochlorogenic acid (99.3%), cryptochlorogenic acid (99.8%), and caffeic acid (98.5%) used in quantification analysis were purchased from Biopurify, Chengdu, China, and chlorogenic acid (99%) from was purchased from Extrasinthese, Genay, France. Chlorogenic acid ≥ 95% (titration) product reference (Ref.) C3878-1G, 3-(4,5-dimethylthiazol-2-yl)-2,5-diphenyltetrazolium bromide (MTT), Wnt CHIR 99021 inductor, and iCRT14 inhibitor were purchased from Sigma-Aldrich, Burlington, MA, USA. Acidified isopropyl alcohol, PBS, fetal bovine serum (FBS), Dulbecco’s modified eagle’s medium (DMEM), Roswell Park Memorial Institute (RPMI) 1640 medium, penicillin, and streptomycin were purchased from GIBCO, Grand Island, NY, USA. The TCF Reporter Plasmid Kit Ref. # 17-285 was purchased from Merck Millipore, Burlington, MA, USA.

### 2.2. Quantification of Chlorogenic Acids and Xanthines by HPLC-DAD

A total of 100 mg of each extract (GC and TC), obtained as previously described [19], were reconstituted with 70% ethanol centrifuged at 13,000 RMP for 10 min; the supernatant was transferred to a 20 mL volumetric flask, and the volume was adjusted. The supernatant was diluted using mobile phase, in proportions of 1 in 20, for the determination of neochlorogenic and cryptochlorogenic acids, and in proportions of 1 in 100 for the determination of chlorogenic acid. For the determination of xanthines, theobromine (99%), caffeine (199.8%), catechin (99%), and epicatechin (90%), the supernatant was diluted using mobile phase, in proportions of 1 in 20. An external standard method was used for quantification in an HPLC Agilent 1200 Series LC System CA, USA, using a Zorbax column SB-C18 from Merck, Burlington, MA, USA.

### 2.3. Cell Culture

Human cell lines SW480 (ATCC, Manassas, VA, USA; CCL-228) and HT-29 (ATCC, Manassas, VA, USA; HTB-38™) derived from colorectal adenocarcinoma, were used. SW480 and HT-29 cells were maintained in Dulbecco’s Modified Eagle Medium (DMEM) and RPMI 1640 medium GIBCO, Grand Island, NY, USA, respectively, supplemented with 10% fetal bovine serum and antibiotics. The cellular passages were made at 70% of cell confluence. The cells were cultured under controlled conditions at 5% CO_2_, 70% humidity, and 37 °C.

### 2.4. Cytotoxicity, Migration, and Invasion Studies

Two coffee extracts rich in green and toasted polyphenols (GC, TC) and CGA were prepared and used as previously reported [19]. In brief, all treatments were dissolved in a culture medium before treating the cells at different concentrations. The cytotoxicity of CGA and coffee extracts was assessed using MTT and SRB assays in 96 well plates at 2 × 10^4^ cells/well. Cell viability was expressed as a percentage of the control as (absorbance of treated cells−absorbance of background controls)/(absorbance of nontreated cells−absorbance of background controls) ×100. For evaluation of cell migration, SW480 and HT-29 cells were seeded in 24-well plates at 4 × 10^5^ cells/well, and a scratch was made with a 10 µL pipette tip, using subtoxic concentrations of 750 µg/mL (GC), 500 µg/mL (TC), and 187 µg/mL (CGA). The images were captured using an inverted microscope (Eclipse Ti-s) with a DsFi1c digital camera from Nikon, Tokyo, Japan (magnification: 10×) at 0, 24, 48, 72, 96, and 120 h. A total of 144 bright-field images were included for each biological replicate, after which, the images were analyzed for segmentation and quantification with Bio-EdiP from ITM, Medellin, Colombia [20]. A scratch in non-treated cells (NTC) was employed to support normal wound healing progression in vitro. For the invasion test, the commercial fluorometric Kit CytoSelect™ 96-well cell invasion assay from Cell Biolabs San Diego, CA, USA was used, treating the 2 × 10^4^ cells/well for 24 h at concentrations of 750 µg/mL, 1500 µg/mL, and 2000 µg/mL for GC; 500 µg/mL, 750 µg/mL, and 1000 µg/mL for TC; and 150 µg/mL, 375 µg/mL, and 750 µg/mL for CGA. Migration and invasion tests were in serum-free media conditions, and bovine serum albumin (BSA) was used as a chemoattractant in the invasion test. The procedure was performed following the manufacturer’s instructions. The suspension of HT-29 and SW480 cells in a culture medium without fetal bovine serum (FBS) was added to the upper chamber and in the lower chamber FBS, it was applied as a chemoattractant. Finally, the fluorescence was read with the fluorescence plate reader (GloMax-Multi Detection System by Promega, Madison, WI, USA) at 480 nm/520 nm.

### 2.5. Wnt Pathway Reporter Assay

For the Wnt pathway reporter assay, SW480 and HT-29 cells were seeded in 12 well plates at 3 × 10^5^ cells/well. After 24 h, cells were transfected in serum and antibiotic-free media with 1 µg of TOP-Flash (plasmid that contains wild type TCF binding sites) or 1 µg of Fop-Flash (plasmid that contains mutated TCF binding sites), both from Millipore, using Lipofectamine^®^ 2000 from Thermo Fisher, Waltham, MA, USA. Cells were treated 24 h after transfection with different concentrations of coffee extracts, CGA, or molecules to control the induction and inhibition of the Wnt pathway, such as CHIR 99021 and iCRT14, respectively. The luciferase activity was determined at 24 h of treatments using the Luciferase Assay System from Promega. The efficiency of transfection was determined by flow cytometry using GFP reporter plasmid. The promoter activity was expressed as the net of TOP-Flash relative light units after the substation of the associated FOP-flash relative light units. All assays were performed in triplicate.

### 2.6. RNA Isolation and RT-PCR

Total RNA was extracted with a Trizol solution (Ref. T9424), from Sigma, Burlington, MA, USA) according to the manufacturer’s instructions. The concentration of RNA was determined by spectrophotometry (Nanodrop 2000/2000C, Thermofisher, Waltham, MA, USA). Two micrograms of total RNA were reverse-transcribed with the RevertAid Reverse Transcriptase kit (Ref. EP0442, Thermofisher, Waltham, MA, USA) with reaction conditions of 25 °C for 6 min, followed by 42 °C for 6 min and 70 °C for 6 min. Finally, cDNA from SW480 and HT-29 cells treated with GC, TC, and CGA for 24 h was stored at −20 °C.

### 2.7. Wnt Target Gene Analysis

For this purpose, quantitative PCR analysis was run in the Thermocycler CFX96 system (Bio-Rad, Hercules, CA, USA) with the Maxima SYBR Green qPCR Master Mix (2X) (Ref. K0252, Thermo Fisher) according to the manufacturer’s protocol. Cycling conditions were 95 °C for 5 min, followed by 40 cycles of 95 °C for 15 s and 60 °C for 60 s. GAPDH was used as an endogenous reference. The 2^−ΔΔCt^ method was used to calculate the differences in the expression levels of CTNNB1, CDH1, and CCND1 during the different treatments. All experiments were run in triplicate to indicate intra-assay variation. The primer sequences were as follows: CTNNB1 forward (F): 5′-TCCGAATGTCTGAGGACAAGC-3′, reverse (R): 5′-CCAAGATCAGCAGTCTCATTCCA-3′; CDH1 (F): 5′-TCCTGGGCAGAGTGAATTTTG-3′, (R): 5′-CTGTAATCACACCATCTGTGC-3′; CCND1 (F): 5′-GAAGATCGTCGCCACCTG-3′, (R): 5′-TCGACATGGAGTCCCAGGA-3′; and GAPDH (F): 5′-AACGGGAAGCTTGTCATCAA-3′, (R): 5′-TGGACTCCACGACGTACTCA-3′.

### 2.8. Subcellular Localization of Wnt Proteins

To determine the subcellular localization of Wnt proteins, cell staining and confocal microscopy were carried out. Briefly, 15 × 10^4^ cells were seeded in 24 well plates with circular glass covers overnight. The next day, the cells were treated with different concentrations of coffee extracts and CGA for 24 h. Afterward, the medium was removed, and cells were fixed in 4% formaldehyde in PBS for 15 min and permeabilized by 0.5% Triton X-100 in PBS for 5 min. After permeabilization, the samples were treated with a blocking solution (Invitrogen, Waltham, MA, USA) for 45 min and then incubated with primary antibodies (against β-catenin and E-cadherin) in TBS overnight. The following day, the cells were washed with PBS and incubated with secondary antibodies from Thermofisher, Waltham, MA, USA). (Alexa-Fluor™488-anti-rabbit and/or Alexa-Fluor™568-anti-mouse IgG) for 45 min. Each sample was washed, mounted, and monitored with an FV1000 Olympus laser scanner confocal microscope from Evident corporation, Tokyo, Japan.

### 2.9. Statistical Analysis

GraphPad 6 was used to perform statistical analysis (GraphPad Software (version number 6)) from GraphPad Software Inc, San Diego, CA. USA. The number of observations represents the categorical data. The variables with normal distributions were denoted by the mean and standard deviation. The variations in cell viability, open wound area, invasion, qPCR data, and immunofluorescence were analyzed using a two-way ANOVA. A *p*-value of ≤0.05 was considered statistically significant.

## 3. Results

### 3.1. Quantification of Chlorogenic Acids, Xanthines, and Catechins

The main metabolites in coffee extracts (Table 1) were determined by HPLC (high-performance liquid chromatography) equipped with a PDA (photodiode-array) detector. GC presented a concentration of chlorogenic acid 4.4 times greater than TC, while neochlorogenic and cryptochlorogenic acids, as well as caffeine and theobromine, showed a slightly higher concentration than TC. None of the studied extracts presented a quantifiable concentration of chatechin or epichatechin.

### 3.2. Cytotoxicity Activity

**Figure 2:**
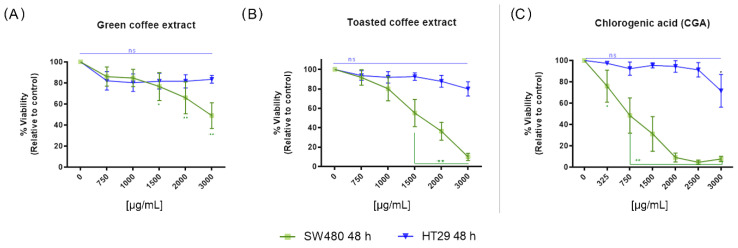
The cytotoxic activity at 48 h measured by MTT in the colorectal cancer cell lines SW480 and HT-29. Treatments with green coffee extract (GC) (**A**), toasted coffee extract (TC) (**B**), and CGA (**C**). Values are expressed as the mean ± SEM of at least three independent experiments. Two-way ANOVA, difference to non-treated cells, * *p* ≤ 0.05, ** *p* ≤ 0.01; ns: non-significant differences.

The cytotoxic activity of GC, TC, and chlorogenic acid (CGA) against SW480 and HT-29 cells was determined by the MTT and SRB methods for 24 and 48 h treatment times (Supplementary Figure S1), and 48 h treatment by MTT (Figure 2). All treatments showed similar cytotoxic activity with the two methods employed and were comparable with a dose/time-dependent tendency to decrease the cell viability. All treatments were more effective in SW480 cells than in HT-29 cells. A significant decrease in cell viability was observed with doses above 1500 µg/mL for GC and TC, and above 325 µg/mL for CGA. Even the highest dose (3000 µg/mL) at 48 h did not cause a significant decrease in cell viability in HT-29 cells. Figure 2A,B shows the difference between the cytotoxicity caused by GC and TC in SW-480 and HT-29 cells. Furthermore, for CGA (Figure 2C), only the highest concentration (3000 µg/mL) significantly decreased the viability of HT-29 cells. All treatments were evaluated by the SRB method (Supplementary Figure S1). Table 2 shows the IC50 values for all the evaluated treatments by MTT and SRB methods in HT-29 and SW480 cell lines. The data for the MTT analysis in SW480 cells were extracted from our previous report [19].

### 3.3. Wound Healing Assay

**Figure 3:**
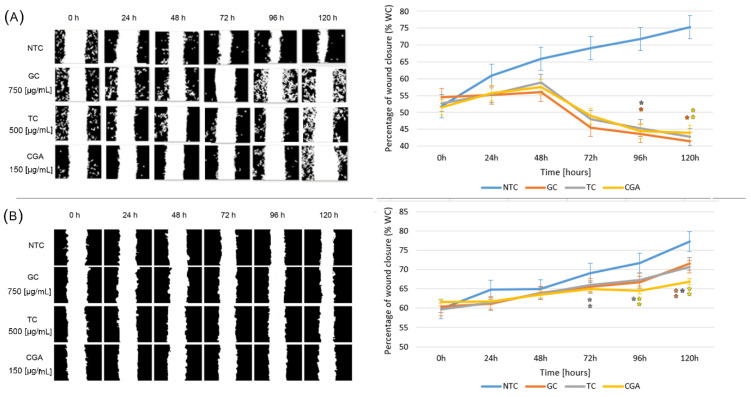
The inhibition of cellular migration on SW480 (**A**) and HT-29 (**B**) cell lines non-treated (blue) and treated with GC (orange), TC (grey), and CGA (yellow). Cell migration was observed with an inverted microscope (10× magnification) at intervals of 24 h over 120 h. Representative images are on the left panel. The right panel shows the quantitative analysis of cell migration by the percentage of wound closure. Values are expressed as the mean ± SEM of three independent experiments. Two-way ANOVA, difference to non-treated cells, * *p* ≤ 0.05 and ** *p* ≤ 0.01.

To determine the effect of treatments on cell migration inhibition, wound healing assays were performed using subtoxic doses of GC (750 µg/mL), TC (500 µg/mL), and CGA (187 µg/mL). Pictures to monitor the healing process were taken every 24 h. The cytotoxic activity results reported in the previous section are in accordance with the wound healing inhibition activity. All treatments have a higher impact on the decrease in the wound closure area in SW480 cells than in HT-29 cells (Figure 3). The wound closure rate was diminished in all treatments in both cell lines, even though the behavior is different. In SW480 (Figure 3A) the wounds healed at a slow rate with respect to the NTC (non-treated cells) during the first 48 h; after which, the wound closure rate decreased, reaching lower values than the initial condition. On the other hand, the results of HT-29 cells (Figure 3B) showed an effect comparable to the control condition, with a tendency to close the wound. CGA treatment significantly inhibited the rate of wound healing, followed by GC and CT, compared with the NTC. The data for the wound healing analysis in SW480 cells were extracted from our previous report [19].

### 3.4. Invasion Assay

The CytoSelect™ 96-well cell invasion assay was used to evaluate the effect of treatments on cell invasion. Three subtoxic doses were selected for each treatment: 750 µg/mL, 1500 µg/mL, and 2000 µg/mL for GC; 500 µg/mL, 750 µg/mL, and 1000 µg/mL for TC; and 150 µg/mL, 375 µg/mL, and 750 µg/mL for CGA. To observe the basal level of invasion of both cell lines, two control conditions were used: with and without chemoattractant, to which no treatment was applied. Finally, the invasive cells that were able to cross the extracellular matrix protein membrane to the lower chamber were quantified with a fluorometric method, where fluorescence values are proportional to the number of invasive cells. The results of the controls showed a greater activation effect for the invasion in response to the chemoattractant in SW480 cells, which agrees with the migratory properties observed before for this cell line.

**Figure 4:**
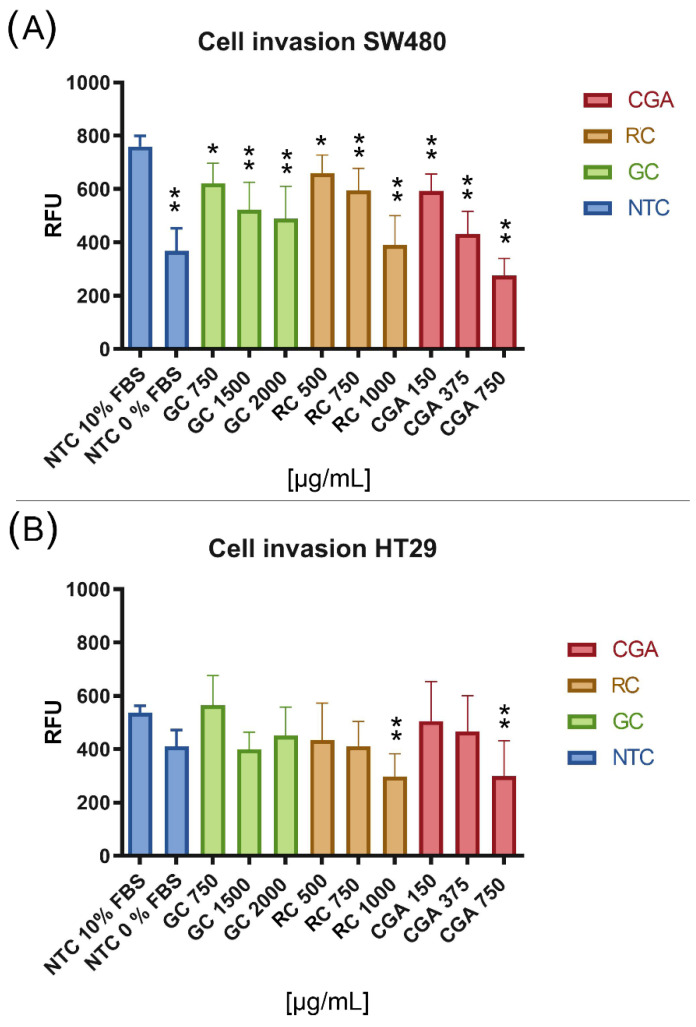
The inhibition of the invasion process of SW480 (**A**) and HT-29 (**B**) cell lines. The percentage of relative fluorescence units (RFU) is proportional to the number of invasive cells. FBS 10% was used as a chemoattractant control and treatment exposure was 24 h. Values are expressed as the mean ± SEM of three independent experiments. Two-way ANOVA, the difference to non-treated cells, * *p* ≤ 0.05 and ** *p* ≤ 0.01.

The results of the effect of the coffee extracts and CGA showed a powerful inhibition of the invasion of SW480 cells compared to the control with chemoattractant (Figure 4A), consistent with the cytotoxic and anti-migratory activities of the treatments on these cells. Additionally, TC 1000 µg/mL had an effect comparable to the control without chemoattractant while CGA 750 µg/mL exceeded the inhibition effect of invasion compared to this control. In contrast, the results for HT-29 cells (Figure 4B) showed a lower effect for the inhibition of cell invasion, considering that only the treatments with higher doses of TC and CGA had a significant impact compared to the control with a chemoattractant. However, these treatments had a higher effect on inhibition of invasion compared to the control without chemoattractant.

### 3.5. Top/Fop Flash Assay

**Figure 5:**
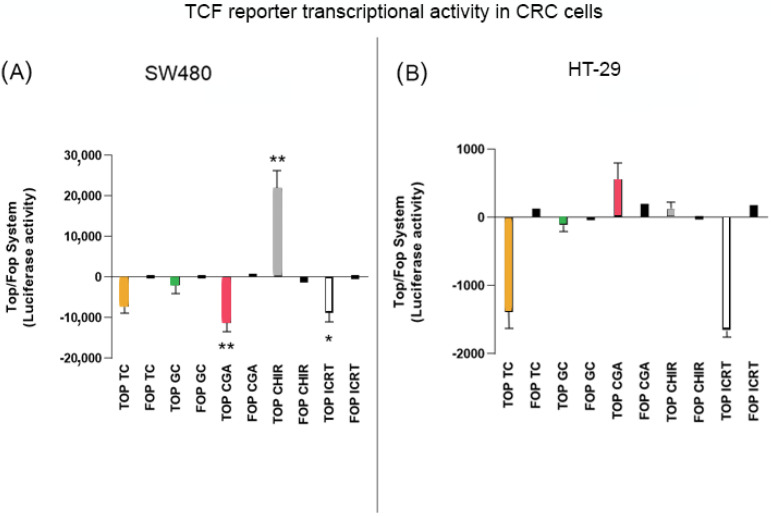
The inhibition of TCF promotor activation of the Wnt pathway plasmid reporter system in SW480 (**A**) and HT-29 (**B**) cell lines. CHIR 99021 was used as a pathway inductor and iCRT14 as an inhibitor. Treatment exposure was 24 h after transfection. Values are expressed as the mean ± SEM of three independent experiments. Two-way ANOVA, difference to non-treated cells, * *p* ≤ 0.05 and ** *p* ≤ 0.01.

To determine if the treatments had an impact on the regulation of transcriptional activity of the Wnt/β-catenin pathway, HT-29, and SW480 cells were transfected with the TOP/FOP Flash reporter plasmid and treated with subtoxic doses of GC (1500 µg/mL), TC (750 µg/mL), and CGA (375 µg/mL) for 24 h. As controls of the regulation of the Wnt pathway, doses lower than the IC50 were employed. For positive and negative regulation of the pathway, the selective inducer CHIR 99021 (10 µM) and the selective inhibitor iCRT14 (40 µM) were included in the experiments. As we expected, the results in SW480 cells (Figure 5A) show a substantial effect on the induction of pathway activation with CHIR, while the inhibition effect of the transcriptional activity of the pathway by iCRT14 is significant compared to the NTC condition. Treatments with GC and TC did not have significant differences in the regulation of the pathway, while CGA had a comparable effect with the selective inhibitor. In the case of HT-29 cells (Figure 5B), the effect of the inducer was lower than in SW480, and treatments with GC, TC, and CGA had a trend of negative regulation of the pathway. However, only TC and CGA had significant values, and CGA had a comparable effect to the selective inhibitor.

### 3.6. Changes in mRNA Expression Levels of CDH1, CTNNB1, and CCND1

**Figure 6:**
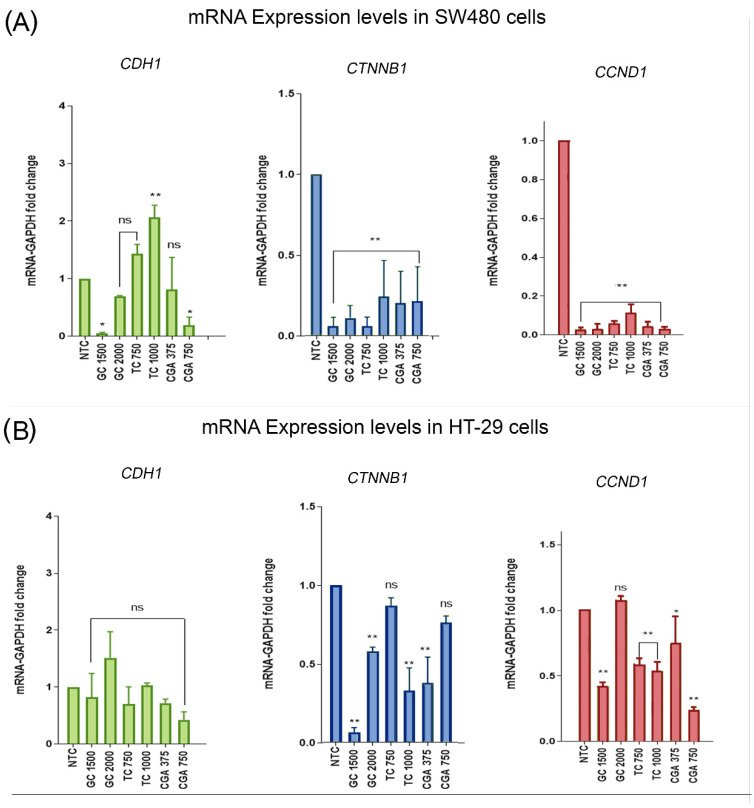
(CGA) Chlorogenic acid, (TC) toasted coffee extract, and (GC) green coffee extract modulation of the mRNA expression levels of *CDH1,* in green bars (which encodes for E-cadherin); *CTNNB1*, in blue bars (which encodes for β-catenin); and *CCND1*, in red bars (which encodes for Cyclin D1) in SW480 (**A**) and HT-29 (**B**) cell lines. Values are expressed as the mean ± SEM of three independent experiments. Two-way ANOVA, difference to non-treated cells, * *p* ≤ 0.05 and ** *p* ≤ 0.01. ns: non-significant differences.

qPCR analysis was used to find possible differences in the mRNA expression levels of genes related to the Wnt/β-catenin pathway, such as *CDH1* (which encodes for E-cadherin), *CTNNB1* (which encodes for β-catenin), and *CCND1* (which encodes for cyclin D1). *GAPDH* was used to normalize expression levels. The treatments included two subtoxic doses of each treatment for 24 h: GC (1500 µg/mL and 2000 µg/mL), TC (750 µg/mL and 1000 µg/mL), and CGA (375 µg/mL and 750 µg/mL). The results are shown in Figure 6.

In the case of E-cadherin in SW480 cells, we found a significant increase in mRNA levels with TC at the highest doses, while GC and CGA mainly had a negative regulatory effect. For HT-29 cells, we observed a tendency for mRNA levels to increase only in GC with 2000 µg/mL. For β-catenin mRNA levels in both SW480 and HT-29 cells, all treatment conditions showed a negative expression regulation effect, being stronger in SW480. Likewise, cyclin D1 expression levels decreased significantly in both cell lines, as expected in accordance with the result found regarding the negative regulation of β-catenin mRNA levels.

### 3.7. Subcellular Localization of β-Catenin and E-Cadherin Proteins

**Figure 7:**
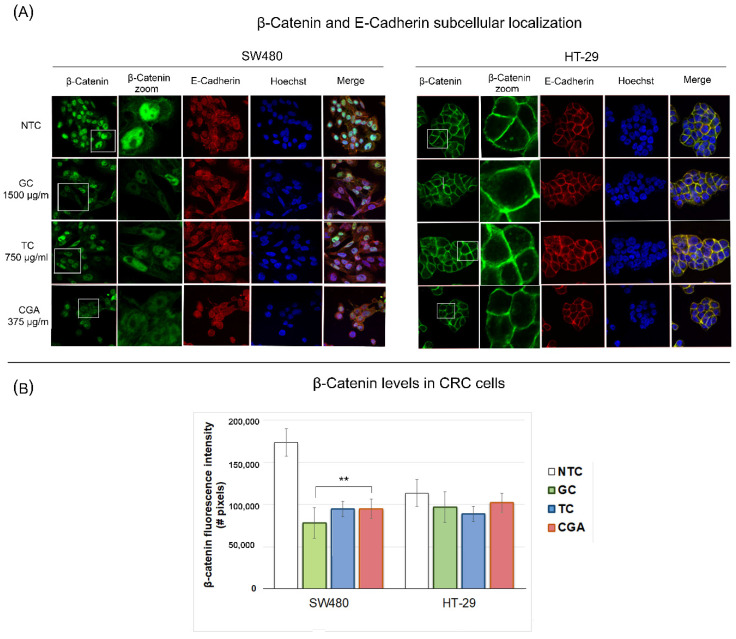
Subcellular localization of β-catenin (green) and E-cadherin (red) proteins in SW480 and HT29 cells treated with chlorogenic acid (CGA), roasted coffee (TC), and green coffee (GC) at 24 h. The panel (**A**) includes representative images, and the (**B**) panel is the β-catenin protein fluorescence intensity in both treated cell lines. The images were captured through an Olympus FV1000 confocal laser scanner microscope with a 60x objective and image scale of 150 µm. Values are expressed as the mean ± SEM of three independent experiments. Two-way ANOVA, difference to non-treated cells, ** *p* ≤ 0.01; ^#^ = number.

Confocal microscopy was performed to determine the effect of the treatments on the subcellular localization of β-catenin and E-cadherin proteins. SW480 and HT-29 cells were exposed for 24 h to GC 1500 µg/mL, TC 750 µg/mL, and CGA 375 µg/mL. The results of the previously described assays showed the increased sensitivity of SW480 cells for all treatments. Consistently with this, we observed that under control conditions without any treatment, the distribution of nuclear β-catenin was significantly different between SW480 and HT-29 (Figure 7). Specifically, the results showed high levels of nuclear β-catenin in SW480 cells, whereas we observed a specific distribution of cytoplasmic and cell membrane β-catenin in HT-29 cells, which correlates with a high percentage of β-catenin/E-cadherin co-localization.

The response to the treatments in SW480 cells (Figure 7) showed a decreased β-catenin fluorescence intensity compared to the control condition, which was most marked in GC, followed by TC and CGA, while the fluorescence intensity levels of E-cadherin remained similar. On the other hand, for HT-29 cells, no nuclear β-catenin staining was observed, and the levels of co-localization between β-catenin and E-cadherin proteins were found to be high in the control condition and maintained in treatments. Thus, during all the treatments, we did not find significant differences in the cellular distribution of β-catenin/E-cadherin, and the β-catenin fluorescence intensity was similar to the control condition.

## 4. Discussion

Phytochemicals and their derivatives are promising options for preventing and treating diseases such as cancer. In vitro and in vivo studies and clinical trials have shown their regulation of various physiological processes, such as inflammation reduction, cell differentiation regulation, and protection against oxidative damage of organelles and DNA [21,22,23]. In recent decades, polyphenols have been highlighted in the context of phytochemicals, and several recent studies have reported on different types of cancer, such as liver, breast, prostate, and CRC [24,25,26].

Diet is the primary source of polyphenols in humans. Coffee, tea, apple juice, and red wine are the most consumed beverages worldwide and represent the main sources of polyphenols in the diet [27,28]. Epidemiological studies on the relationship between colorectal cancer and coffee consumption are inconclusive because results vary by study design, cancer site, gender, and ethnicity. However, current evidence suggests an inverse relationship in case-control studies where the category of high coffee consumption is associated with a 15% to 21% decreased risk of colon cancer [29]. Among the bioactive components of coffee, which include caffeine, melanoidins, and diterpenes, the most abundant polyphenols are chlorogenic, caffeic, and ferulic acids, the levels of which may vary depending on the coffee species, degree of roasting, and preparation technique [30].

Exposure to coffee polyphenols could promote colorectal cancer chemoprevention through diverse mechanisms, such as the activation of anti-mutagenic pathways, modification of the microbiome, or modulation of signaling pathways critical for cancer development [31]. The Wnt pathway plays a critical role in the carcinogenesis and progression of colorectal cancer. Recent studies show the ability of some polyphenols to regulate the Wnt pathway, suggesting the potential of these molecules as a chemopreventive treatment or chemotherapeutic alternative against CRC [32]. However, the involvement of coffee polyphenols in the modulation of the Wnt pathway has not been explored in CRC models.

The present study evaluated the effect of two coffee extracts rich in polyphenols as well as the independent effect of CGA, the most abundant polyphenol in coffee. SW480 and HT-29 colorectal cancer cells were treated to analyze the effect of these using cellular and molecular approximations, such as cytotoxic potential, migration, invasion modulation, and Wnt pathway transcriptional activity.

In the first instance, MTT and SRB analyses were carried out to determine the cytotoxic effect of green coffee extract (GC), toasted coffee extract (TC), and CGA. The SRB method was used because the mechanism of action of the MTT method is related to the reduction capacity of mitochondrial enzymes, and there are reports of interference caused by some polyphenols due to their antioxidant activity [33]. On the other hand, the metabolic activity of MTT reduction can differ between cell lines and be related to the induction of injury and cell death. At the same time, the SRB method, based on binding to slightly acidic total proteins, is considered a complementary test for determining the cytotoxic potential [34,35]. In this way, we obtained a complete perspective of the effects of the evaluated treatments. Both methods show a similar trend of decreased cell viability which is more evident in SW480 cells. This trend is most marked in the treatments with CGA, followed by TC and GC (Table 2). However, the MTT (Figure 2) method showed minor IC50 values in the evaluated cell lines that could be related to the mechanism of action, perhaps mainly to metabolic pathways.

TC treatment showed a higher cytotoxic effect in this study than in GC (Figure 2). During the roasting process, the number of polyphenols such as CGA in coffee diminished, and flavonoids increased [30]. The quantification results demonstrated this effect of the roasting process, showing that CGA levels decreased in TC, and xanthine, theobromine, and caffeine increased (Table 1). These conditions, together with the generation of new bioactive molecules such as melanoidins during the roasting process [36,37], could be related to the biological effect of TC.

The IC50 values for CGA in the CRC cell lines found in this study were higher than in previous reports. However, the highest sensitivity continues to be found in SW480 cells, as in the report of Aires et al. where the exposure of HT-29 and SW480 cells to resveratrol for 72 h resulted in IC50 values of 70 µM and 40 µM, respectively [38]. On the other hand, quercetin showed similar behavior, with IC50 values of 75 µM for HT-29 cells and a 70% inhibition of viability with a dose of 80 µM in SW480 cells [39,40].

This effect was evident in the wound healing cell migration assay, where a more significant effect on migration inhibition was observed in SW480 cells with treatments at subtoxic doses (Figure 3A). The effect was comparable in all treatments, where we found a migration inhibition behavior up to 120 h of follow-up. In HT-29 cells (Figure 3B), the effect observed on migration inhibition was less pronounced than in SW480, and CGA had a higher inhibition. Reports on the migration inhibition activity of curcumin on these cell lines showed that HT-29 cells are less sensitive than SW480 cells [41]. Another related report showed the same effect on SW480 cells compared to DLD1 cells, where resveratrol decreased the migration capability, mainly in SW480 [42]. In cell invasion experiments, the behavior was similar, considering that we found the treatments to have a more powerful effect in SW480 cells, where the inhibition of cell invasion was dose-dependent, and the effect of treatments was notable and comparable to the control without chemoattractant (0% SFB) (Figure 4A). The experiments showed that the invasive capabilities of HT-29 cells are less significant than those of SW480, even with chemoattractant (10% SFB); in addition, the treatments with significant differences in decreasing the invasion were CGA and TC (Figure 4B).

After observing the biological effect of treatments on the evaluated in vitro models, differences were found at the cytotoxic level and in the modulation of cell migration and invasion capabilities. Therefore, we performed some experiments to determine whether these effects could be related to the modulation of the Wnt pathway. For this, we initially used the reporter assay system Top/Fop flash, which uses a plasmid that includes the TCF transcription factor sequence. The increase and translocation of β-catenin to the nucleus implies the formation of the β-catenin/TCF complex, inducing the expression of target genes of the Wnt pathway [43]. The results obtained (Figure 5) show that decreasing the transcriptional activation of the plasmid has an effect that is more pronounced with the CGA treatment compared with the specific inhibitor iCRT14 in both cell lines, except in the case of HT-29 cells where the effect of CGA was comparable with TC. Previous reports evaluated the levels of the basal intrinsic activation of the Wnt pathway using the Top/Fop flash system and showed a change up to 19-fold higher in Wnt pathway activation in SW480 vs. HT-29 cells [44]. Meanwhile, another report described a difference of 1.7 for HT-29 to 52.1 for SW480 [45]. This difference in the level of basal activity of the Wnt pathway between cell lines could explain the different effects of the Wnt inductor CHIR and the response to treatments where the induction of transcriptional activity was much higher in SW480 cells only. In addition, the effect on the decrease in transcriptional activity was evident and comparable with other studies that report the effect of some polyphenol extracts and compounds on CRC cells, i.e., the HS7 fraction of the TC. These include camphoratus extract, which showed an inhibition effect on the Wnt pathway on SW480, HCT116, and HT-29 cells, decreasing the transcriptional activity by between 50 and 60% [46]. Another report showed that silibinin significantly inhibits the activity of the pathway at 24 h of treatment with 100 µM in SW480 cells [47].

The regulation of the Wnt pathway was explored with a reporter assay. We performed qPCR experiments to determine the influence of the treatments on the expression of genes related to the pathway, such as CTNNB1 (which encodes for β-catenin), CDH1 (which encodes for E-cadherin), and the Wnt pathway target gene CCND1 (which encodes for cyclin-D1). The results showed that CDH1 expression levels increase in SW480 cells with TC at 1000 µg/mL and decrease with GC and CGA (Figure 6). In HT-29 cells, all treatments had significant differences. For CTNNB1 and CCND1, a significant decrease in the expression levels was observed, particularly in SW480 cells, while for CTNNB1 mRNA levels in HT-29 cells, some doses of treatments with TC and CGA had no significant differences in comparison with the control. A previous report showed the basal expression level of the *CTNNB1* gene on the CCD18-CO normal colon fibroblast and SW480, Caco-2, and HT-29 colorectal cancerous cells. *CTNNB1* was downregulated in HT-29 and Caco-2 cells, and the levels in SW480 and CCD18-CO were higher and similar. Previous studies showed the effect of natural compounds on regulating the expression of these genes related to the pathway. For example, a study reported the effect of two metabolites of ellagitannins, MPhA and MPhb in Caco-2 and CCD18-CO cells, where the treatments did not alter the expression level of *CTTNB1* in either cell line [48]. Another study reported the effect of lupeol on *CTNNB1* expression levels in SW480 and HCT116 after 24 h of treatment, where the expression was significantly downregulated only in HCT116 cells at the higher doses of 80 µM; however, the protein levels in SW480 also decreased. For cyclin D1 in SW480 cells, the gene expression was downregulated from doses of 40 µM and 20 µM in HCT116 cells [49]. In addition, the downregulation of *CTNNB1* and *CDH1* gene expression was significant at 24 h in HT-29 cells, using doses of 120 µM and 50 µM of phenethyl isothiocyanate and sulforaphane, the major isothiocyanates of broccoli [50].

On the other hand, a series of studies report the effect of some natural compounds on the expression of cyclin D1 in models of colorectal cancer, such as diospyros kaki thunb (DKC), which decreased protein and mRNA levels at concentrations of 50 µg/mL, mainly in SW480 cells, after 24 h [51]. In addition, safflower seed (*Carthamus tinctorius* L.) was recently shown to decrease the expression of cyclin D1 mRNA at concentrations of 100 µg/mL at 24 h, with LoVo and HT-29 cells being more sensitive to treatment compared to SW480 and HCT116 [52].

Finally, we performed immunostaining experiments to determine the location at the subcellular level of E-cadherin and β-catenin. For HT-29 cells (Figure 7), we did not observe changes in the subcellular location or co-localization of these proteins with the treatments, with β-catenin mainly in the membrane. On the other hand, in SW480 cells, it was possible to show that the treatments had the effect of reducing the amount of β-catenin, in addition to increasing the amount of β-catenin/E-cadherin co-localization, confirming a potential modulatory effect on the Wnt pathway. Very few studies on polyphenols have addressed the regulation of the Wnt pathway through fluorescence microscopy techniques. One of these few studies showed the effect of some synthetic derivatives of sibylline, where concentrations of 4 µM for 24 h increased the levels of E-cadherin and decreased β-catenin in HCT116 cells [53].

In summary, in the present work, we observed a biological effect of chlorogenic acid and polyphenol-rich coffee extracts on cell migration and invasion, possibly related to transcriptional regulation of the Wnt pathway, which was most pronounced in SW480 cells. The difference in the sensitivity of this cell line compared to HT-29 cells, which were less sensitive, could be related to aspects such as the genetic background of each cell line, which influences the activity levels of the Wnt pathway, and the different protein levels, which are related to transport and detoxification processes.

First, the genetic and epigenetic background of SW480 promotes a higher activation of the Wnt pathway than HT-29. The effect of the treatments on the modulation of the Wnt pathway would be more evident in the cell line with the highest activation of the pathway. The consensus molecular subtypes (CMS) of CRC establish four molecular subtypes based on gene expression profiles that have specific implications in the clinical context, independent of the stage of the disease [54]. This classification is influenced mainly by the tumoral microenvironment; however, in vitro models could show diverse CMS. Colorectal cell lines have been used to identify multiple and specific molecular aberrations for all CMS of CRC. In this context, a multi-omics study established that the HT-29 cell line in the CMS3 metabolic type is related to tubulovillous adenomas with serrated features and more prevalent KRAS mutations. The SW480 cell line was classified in the CMS4 mesenchymal class, a relatively aggressive phenotype related to serrated adenomas, high levels of TGF-β, and very high somatic copy number alteration (SCNA), and are known to be pro-inflammatory and pro-angiogenesis. In the clinical context, these classifications are determined at advanced stages [55]. Mutations of the APC protein indicate the difference in the Wnt pathway activity levels in both evaluated cell lines. APC is truncated at the carboxyl-terminal end at residue 1338 in SW480 and residue 1555 in HT-29 [56]. APC truncated mutations in SW480 compromise some crucial domains in APC, such as the β-catenin inhibitory domain (CID). This has high relevance for β-catenin targeting for ubiquitination and also plays a role in the interaction between the SAMP binding site in APC with axin for the degradation complex, conformation, and stabilization [57]. The reduction in APC truncated protein through RNA interference technology decreases the proliferation of six types of colorectal cancer cells and decreases tumor growth in vivo [57]. On the other hand, another study suggests mechanisms that explain how the truncated APC with CID loss promotes β-catenin deubiquitination by the reverse binding of β-TrCP and USP7 [58].

The implications of APC for the regulation of phosphorylation, ubiquitination, degradation, nuclear transport of β-catenin, and the affectation levels of the APC protein are reflected in the Wnt pathway activation, where these mutations imply inhibition of the degradation of β-catenin in SW480 but not in HT-29, DLD-1, and the wild type APC HCT116 cells.

Secondly, protein levels related to the transport of substances, mainly export, as well as cell detoxification processes between the cell lines evaluated, were reported, including high levels of *MDR1* (multidrug resistance gene) in SW480 and an undetectable protein level of WB in HT-29 cells [38]. The MDR1 coding by the *ABCB1* gene is a member of the superfamily of ATP-binding cassette (ABC) transporters, and the inhibition effect of polyphenols has been reported [59]. Moreover, the BCRP (breast cancer resistance protein) detected in HT-29 cells works as an efflux pump with broad substrate recognition [60] and facilitates the detoxification or expulsion of the treatments. This acts to reduce its effects, while a dynamic of decreased levels of MDR1 in SW480 cells due to polyphenol treatments favors its effect by increasing the intracellular amounts.

Furthermore, some studies have reported different levels of UDP-glucuronosyltransferases (UGTs) in colorectal cancer cells. These UGTs are involved in the glucuronidation process that facilitates the elimination of substances in cells, including phenolic compounds. Low levels of UGT proteins are associated with high drug-induced toxicity, and, in contrast, a high level of UGT is related to the loss of the bio-availability of treatments, premature glucuronidation, and lack of efficiency [61]. In this context, different isoforms of UGT1A are expressed in HT-29 cells and not in HCT116, influencing the intracellular accumulation of tanshinone IIA (TSA), a phytochemical from the Chinese medical herb Salvia miltiorrhiza bunge (danshen), and reducing its antitumoral effect in HT29 cells [62]. On the other hand, HT-29 cells are resistant to treatment with ganetespib, a specific inhibitor of HSP90, while SW480 and HCT116 cells are susceptible to this treatment. Another study evidenced a high correlation between IC50 levels and UGT1A expression, and this effect was reverted with UGT1A knockdown siRNA-mediated in HT29 cells [63].

In this study, we were able to detect the activation levels of the Wnt pathway in both cell lines by analyzing the control conditions, such as cell migration and invasion. The Wnt pathway has been reported to be related to tumor progression, promoting migration and invasion processes. The behavior of these phenomena is higher in untreated SW480 cells [64]. The invasion capability of multiple CRC cells was reported, and the difference in the number of invasive cells was 49.7 for HT-29 compared to 169.5 for SW480. In addition, the level of expression of proteins related to the invasion process, such as vimentin, *N*-cadherin, and ZEB1, was very high in SW480 and low in HT-29 cells [65]. Similarly, in the reporter assay for the evaluation of transcriptional activity, we observed that the specific inhibitor of GSK-3β, the Wnt inducer CHIR, was much more effective in SW480 cells. In contrast, in HT-29 cells, the activation level of the pathway was lower with the inductor treatment, suggesting a possible mechanism of phosphorylation-ubiquitination degradation different from HT-29. This process could be related to the previously-mentioned truncated APC protein, where APC mutations present in SW480 compromise the β-catenin inhibitory domain (CID) [58,66]. In addition, in the controls of the immunostaining tests, the difference in the quantity and subcellular location of β-catenin between both cell lines was evident. We observed a more significant presence of nuclear β-catenin exclusively in SW480 cells. Furthermore, our results showed that coffee polyphenol extracts and CGA reduce the fluorescence intensity of β-catenin in SW480 cells and downregulate the cyclin D1 expressions, suggesting a potential Wnt/b-catenin pathway modulation. This pathway has been established as an important therapeutic target in CRC, and natural and synthetic molecules have been established as modulators and drug candidates for prevention and treatment strategies [67].

## 5. Conclusions

The study of the effect of bioactive molecules on natural products has been of great interest, particularly regarding the polyphenols present in the diet and their effect on CRC and the Wnt pathway. In the present study, the effect of two coffee extracts rich in polyphenols (green coffee and toasted coffee) and CGA on the inhibition of cell viability and modulation of the migratory and invasive properties of SW480 and HT-29 CRC cells was demonstrated. This effect was also accompanied by the potential modulation activity of the Wnt/β-catenin pathway, evidenced through a decrease in the expression of related genes, *CTNNB1* (which encodes for β-catenin), CDH1 (which encodes for E-cadherin), and the Wnt pathway target gene *CCND1* (which encodes for cyclin-D1). In addition, decreased reporter activation for the TCF4 promoter and changes in β-catenin protein fluorescence levels were observed. For all treatments, SW480 cells had a higher sensitivity than HT-29 cells, and the activity of CGA treatments was more evident, followed by TC and GC. These differences in sensitivity between cell lines could be related to the level of mutations in the *APC* gene, which are in turn directly related to the regulation of β-catenin and Wnt pathway modulation. In addition, the differences in the expression profiles of proteins involved in the cellular detoxification of polyphenols contribute to the higher impact of treatment on SW480 cells. Our results provide an exciting starting point on the effect of polyphenols in coffee in the context of CRC and the Wnt pathway. More studies are necessary to determine the specific regulatory mechanism of these molecules on the pathway and the possible implications of the metabolism and the interaction with the microbiota in the in vivo context.

## Figures and Tables

**Table 1:** The chlorogenic acids, xanthines, and catechins in green and toasted coffee extracts (GC, TC) by HPLC-DAD. All analyzes were performed on three independent samples. ND, not detectable. NA, not applicable. RSD, relative standard deviation.

Chlorogenic Acids	Neochlorogenic Acid		Chlorogenic Acid		Cryptochlorogenic Acid		Caffeic Acid	
	mg/100 g sample	RSD	mg/100 g sample	RSD	mg/100 g sample	RSD	mg/100 g sample	RSD
**Green coffee**	1114.50	1.205	17,715.79	4.451	2025.56	6.614	ND	NA
**Toasted coffee**	1485.62	2.023	3996.50	2.087	2095.93	1.929	ND	NA
**Xanthines and catechins content**	**Theobromine**		**Caffeine**		**Catechin**		**Epicatechin**	
	mg/100 g sample	RSD	mg/100 g sample	RSD	mg/100 g sample	RSD	mg/100 g sample	RSD
**Green coffee**	406.51	7.599	2878.03	6.225	ND	NA	ND	NA
**Toasted coffee**	563.84	2.796	3372.86	1.860	ND	NA	ND	NA

**Table 2:** Half maximal inhibitory concentration (IC50) values by MTT and SRB methods on SW480 and HT-29 cells treated with green and toasted coffee extracts (GC, TC) and chlorogenic acid (CGA).

IC _50_ Value by MTT	SW480		HT-29	
	24 h	48 h	24 h	48 h
**Green coffee**	4325 µg/mL	2555 µg/mL	17,715 µg/mL	8416 µg/mL
**Toasted coffee**	3922 µg/mL	2226 µg/mL	9918 µg/mL	13,247 µg/mL
**CGA**	686.6 µg/mL	598.3 µg/mL	8114 µg/mL	6733 µg/mL
**IC _50_ Values by SRB**				
	**24 h**	**48 h**	**24 h**	**48 h**
**Green coffee**	4676 µg/mL	2799 µg/mL	129,197 µg/mL	48,366 µg/mL
**Toasted coffee**	3656 µg/mL	1590 µg/mL	58,901 µg/mL	16,484 µg/mL
**CGA**	2844 µg/mL	1338 µg/mL	72,945 µg/mL	18,379 µg/mL

## Data Availability

Not applicable.

## Supplementary Materials

The following supporting information can be downloaded at: https://www.mdpi.com/article/10.3390/nu14224880/s1, Figure S1: Cytotoxicity activity measured by MTT (A) and SRB (B) in the colorectal cancer cell lines SW480 and HT-29 at 24–48 h.
