# Supplementary material for: Modulatory Effect of Chlorogenic Acid and Coffee Extracts on Wnt/β-Catenin Pathway in Colorectal Cancer Cells
Source: Nutrients. 2022 Nov 18;14(22):4880. doi: 10.3390/nu14224880 (PMC9693551; doi:10.3390/nu14224880)
Supplement: Supplementary file 1 [file nutrients-14-04880-s001.zip › nutrients-2022423-supplementary.pdf]

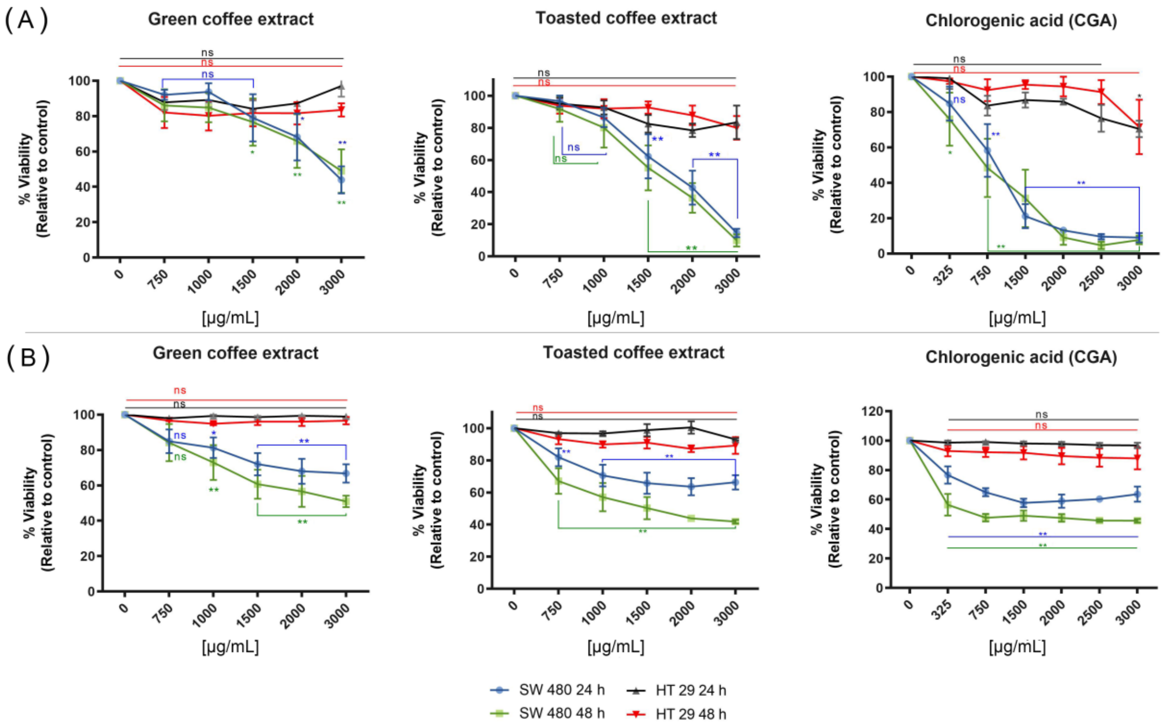

Supplementary Figure S1. Cytotoxicity activity measured by MTT (**A**) and SRB (**B**) in the colorectal cancer cell lines SW480 and HT-29 at 24-48 h. Values are expressed as mean ± SEM of at least three independent experiments. Two-way ANOVA, difference to non-treated cells, * $p \leq 0.05$, ** $p \leq 0.01$.
